# Proteaphagy is specifically regulated and requires factors dispensable for general autophagy

**DOI:** 10.1016/j.jbc.2021.101494

**Published:** 2021-12-14

**Authors:** Kenrick A. Waite, Alicia Burris, Gabrielle Vontz, Angelica Lang, Jeroen Roelofs

**Affiliations:** 1 Department of Biochemistry and Molecular Biology, University of Kansas Medical Center, Kansas City, Kansas, USA; 2 Molecular, Cellular, and Developmental Biology Program, Division of Biology, Kansas State University, Manhattan, Kansas, USA; 3 Biology & Environmental Health, Missouri Southern State University, Joplin, Missouri, USA

**Keywords:** proteasome, autophagy, protein degradation, target of rapamycin (TOR), proteaphagy, starvation, proteasome inhibitor, vacuole, yeast, CP, core particle, RP, regulatory particle, TORC1, target of rapamycin complex 1, UPS, ubiquitin-proteasome system, YPD, yeast-peptone dextrose

## Abstract

Changing physiological conditions can increase the need for protein degradative capacity in eukaryotic cells. Both the ubiquitin-proteasome system and autophagy contribute to protein degradation. However, these processes can be differently regulated depending on the physiological conditions. Strikingly, proteasomes themselves can be a substrate for autophagy. The signals and molecular mechanisms that govern proteasome autophagy (proteaphagy) are only partly understood. Here, we used immunoblots, native gel analyses, and fluorescent microscopy to understand the regulation of proteaphagy in response to genetic and small molecule-induced perturbations. Our data indicate that chemical inhibition of the master nutrient sensor TORC1 (inhibition of which induces general autophagy) with rapamycin induces a bi-phasic response where proteasome levels are upregulated after an autophagy-dependent reduction. Surprisingly, several conditions that result in inhibited TORC1, such as caffeinine treatment or nitrogen starvation, only induced proteaphagy (i.e., without any proteasome upregulation), suggesting a convergence of signals upstream of proteaphagy under different physiological conditions. Indeed, we found that several conditions that activated general autophagy did not induce proteaphagy, further distinguishing proteaphagy from general autophagy. Consistent with this, we show that Atg11, a selective autophagy receptor, as well as the MAP kinases Mpk1, Mkk1, and Mkk2 all play a role in autophagy of proteasomes, although they are dispensable for general autophagy. Taken together, our data provide new insights into the molecular regulation of proteaphagy by demonstrating that degradation of proteasome complexes is specifically regulated under different autophagy-inducing conditions.

Two major pathways for eukaryotic cells to recycle proteins are lysosomal targeting and the ubiquitin-proteasome system (UPS). Within the UPS, E3 ubiquitin ligases recognize substrates destined for degradation. The E3 ligases act in concert with ubiquitin-conjugating enzymes to modify lysine residues of the substrate with ubiquitin or a chain of ubiquitins. Ubiquitinated substrates are often targeted for degradation with the ubiquitin chain being recognized by the proteasome. In a complex process, the proteasome unfolds substrates, removes the ubiquitin moiety, and hydrolyzes the substrate, producing short peptides. The short peptides are further processed by peptidases into amino acids that become intermediates for various metabolic processes (1, 2). Lysosomes receive substrates either from endocytosis (extracellular and plasma membrane components) or through autophagy (intracellular components). Autophagy, in this article referring to macroautophagy, uses a process where the substrates are engulfed into a double membrane compartment known as the autophagosome (3, 4, 5). The targeting of substrates to autophagosomes, like the UPS, is often initiated by ubiquitination of target proteins. The ubiquitinated proteins are recognized by autophagy adapters, such as p62/SQSTM1 in humans and Cue5 in yeast, that bind LC3 (humans) or Atg8 (yeast) found on expanding preautophagosomes (6, 7, 8, 9). The binding is mediated by LC3-interacting regions or Atg8-interacting motifs in the autophagy adapters. Closure of the preautophagosome captures the substrates. The formed autophagosome then fuses with lysosomes (or vacuoles in yeast and plants) and the content is degraded, producing amino acids. Specific transporters in the lysosomal membrane export the amino acids to the cytosol (10). The ability of both the UPS and lysosomal degradation to contribute to the amino acid pool in cells becomes particularly important during physiological conditions that reduce amino acid levels. For example, under the conditions of nitrogen starvation, autophagy becomes essential and is the major route for amino acid recycling (11, 12).

Autophagy and the UPS both replenish the amino acid pool as well as use ubiquitination as a signal for degradation. Therefore, it makes sense that these degradative pathways are coordinated and have some level of redundancy. Indeed, the upregulation of one pathway can, to some extent, compensate for the impairment of the other (13, 14, 15). For example, proteasome inhibitors have been shown to induce an autophagic response, and mTOR inhibition by rapamycin, which induces autophagy, was shown to relieve proteotoxic stress caused by proteasome inhibition (16, 17). Further, an increase in proteasome activity was reported in yeast and mammalian cells deleted for *ATG5, a gene* essential for autophagy, as well as upon chemical inhibition of autophagy (18). Similarly, *atg32Δ* cells upregulate proteasome activity to compensate for an inability to induce nonselective autophagy upon antimycin A treatment (18). Some functions are however clearly unique to each pathway. Proteasomes are, in large, responsible for the degradation of short-lived proteins like IκB (during NFκB signaling) and cyclins (during cell cycle progression) (19, 20). Autophagy, on the other hand, can dispose off aggregated proteins or defective (parts of) organelles directly through the extension of autophagic membranes.

Autophagy is induced by a number of cellular stresses such as nutrient starvation, mitochondrial dysfunction, and various infections (3, 10, 21). This process can capture cytosolic material nonspecifically and be selective for specific cargo. Examples of the latter are mitophagy, pexophagy, and ER-phagy, where unique receptors (Atg32, Pex14, and Atg39/40 respectively) ensure specific and efficient targeting to autophagosomes (4, 22, 23, 24). Besides organelles, selective autophagic degradation has also been observed for ribosomes in yeast and mammalian cells, a process named ribophagy (24, 25, 26, 27). Proteasomes are another multisubunit complex that can be targeted for autophagic degradation, a process referred to as proteaphagy. In yeast, nitrogen starvation and proteasome inhibitor treatment induce proteaphagy (28, 29). The process is conserved as it has also been observed in *Arabidopsis* and human cells (30, 31, 32). Further, proteaphagy appears to be selective as receptors targeting proteasomes to autophagosomes have been identified. For example, p62 upon amino acid starvation in mammalian cells (30), and Rpn10 and Cue5 upon proteasome inhibition in plants and yeast respectively (29, 31). These receptors bind Atg8 (or LC3 in mammals), which facilitates proteaphagy. Furthermore, several factors that are dispensable for bulk autophagy are important for proteaphagy upon nitrogen starvation in yeast (28, 31, 33). How proteaphagy is regulated, however, remains poorly understood.

The target of rapamycin complex 1 (TORC1) is a master regulator-controlling cell growth and metabolic activity based on the cell's physiological state. Under nutrient-rich conditions, TORC1 is active, and general autophagy is inhibited through the phosphorylation and inactivation of Atg13 and Atg1/Ulk1 (34, 35, 36). Interestingly, treatment of cells with the TORC1 inhibitor rapamycin has been shown to increase proteasome levels in yeast and mammalian cells (34, 37, 38). Further, an increase in K48-linked ubiquitinated substrates has been observed, along with an increase in proteasomal degradation of substrates in mammalian cells treated with mTOR inhibitors. Apparently, the capacity of protein degradation by proteasomes becomes important under conditions where TORC1 is not active. Surprisingly, proteasomes undergo autophagic degradation in yeast, plants, and mammalian cells upon starvation conditions well-known to cause TORC1 inhibition (28, 30, 31). To better understand the response of proteasomes to autophagic stimuli, we sought to determine how proteaphagy was regulated in yeast. We used various chemical treatments and physiological conditions known to induce general autophagy. Our data show a biphasic response upon inhibition of TORC1 with rapamycin. During the first 4 h of treatment, proteasome levels and activity increased. After this, proteasomes were targeted for vacuolar degradation. However, nitrogen starvation, which results in TORC1 inhibition, did not induce a similar response. Indeed, our data show that various stimuli that cause TORC1 inhibition and induce general autophagy, showed little to no proteaphagy. Thus, proteaphagy is regulated distinctly from general autophagy. Consistent with this, we identified several proteins required for proteaphagy, namely the regulatory kinases Mpk1, Mkk1, and Mkk2 as well as the selective autophagy receptor Atg11.

## Results

### Rapamycin induces a bi-phasic proteasome response

**Figure 1:**
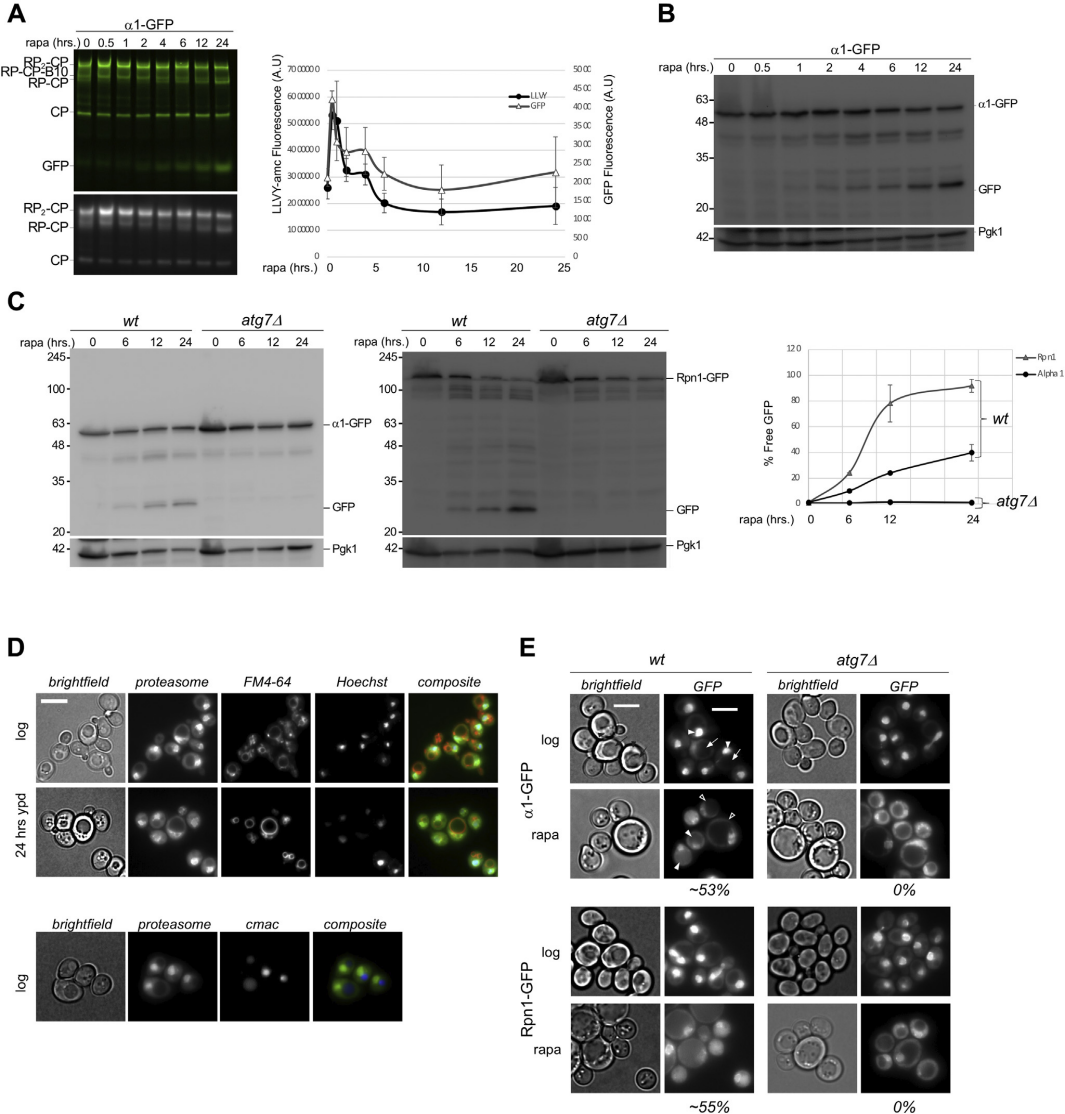
**Rapamycin induces a bi-phasic proteasome response.***A,* yeast expressing α1-GFP were grown in YPD medium and treated with 200 nM rapamycin. Equal volumes of lysates were separated on native gels and imaged for GFP (*top panel*) or peptidase activity in the presence of 0.02% SDS using the proteasome substrate suc-LLVY-AMC (*lower panel*). The graphs show the quantifications of the levels of GFP and LLVY peptidase activity corresponding to the RP_2_-CP species on native gel, both normalized using Pgk1 signal from three experimental replicates. The error bars represent SEM. *p* values for upregulation at the half hour timepoint were 0.006 for GFP and 0.03 for LLVY-AMC using unpaired *t* test. *B,* the samples treated as in (*A*) were denatured and separated on SDS-PAGE and immunoblotted for GFP. The *upper band* shows α1-GFP and *lower band* is “free” GFP resulting from α1-GFP cleavage by vacuolar hydrolysis. Pgk1 was used as a loading control. The data presented are representative of three independent experiments. *C,* WT and *atg7*Δ yeast expressing α1-GFP or Rpn1-GFP were inoculated in YPD medium at A_600_ of 0.5 and grown to log phase (∼4 h). The cells were treated with rapamycin as in (*A*) and lysed at indicated time points. α1-GFP, Rpn1-GFP, and cleaved free GFP (indicative of vacuolar targeting) were monitored by immunoblotting for GFP. Pgk1 was used as a loading control. The graph shows quantification of the immunoblots from 2 independent experiments and the error bars represent SEM (*D*) Rpn1-GFP expressing yeast was stained for the vacuole membrane using FM4-64 and the nucleus using Hoechst 33,342. Microscopy was performed at log phase and following 24 h growth in rich media (YPD) (*top*). Rpn1-GFP expressing yeast growing logarithmically were incubated with the vacuole lumen marker CMAC-arg (*bottom*). The scale bars represent 0.5 μm and data presented are representative of consistently observed staining patterns from many independent experiments (*E*) microscopic analysis of yeast collected at log phase and 24 h after rapamycin treatment. In *top* image, the *arrowheads* point to nuclei and *arrows* to vacuoles. In rapamycin-treated cells, the filled *arrowheads* indicate cells with vacuolar fluorescent signal, whereas open *arrowheads* show subset of nonresponding cells. The scale bars represent 0.5 μm. The values indicate the percentage of cells in WT and *atg7Δ* with vacuolar GFP signal after rapamycin treatment. Vacuolar GFP signal was rarely (<1 %) observed in nontreated cells. Approximately, 55% of WT cells showed vacuolar localization of proteasomes after rapamycin treatment (n > 100, SEM= 1.3 from three independent experiments). CP, core particle; RP, regulatory particle; YPD, yeast-peptone dextrose.

Upon nitrogen starvation, proteasomes are targeted for vacuolar degradation via an autophagy dependent pathway. The extent of this degradation appears to vary (28, 31, 33). To elucidate the signaling pathways involved, we sought to determine the role of TORC1 inhibition in this process as TORC1 inhibition occurs upon nitrogen starvation and induces autophagy. However, the TORC1 inhibitor rapamycin has been reported to increase proteasome levels and activity (38, 39). To study this contradiction, we monitored the response of proteasomes to rapamycin treatment over an extended time by native gel analyses of yeast lysates. We introduced the sequence of eGFP before the stop codon of the core particle (CP) subunit α1 or regulatory particle (RP) subunit Rpn1. This was performed at the endogenous locus to ensure that transcription is regulated as in WT, and no untagged subunits are produced (40). Native gel analyses comparing proteasome activity in the presence of 0.02% SDS in untagged versus GFP-tagged strains showed similar activity and rapamycin response. Because SDS opens the CP gate, thus eliminating the effect of other regulators of proteasome activity, this result indicates that GFP tagging alone is unlikely to cause a change in proteasome abundance (Fig. S1). Upon treating cells with rapamycin, a statistically significant upregulation of 26S proteasome (RP2-CP) was observed as determined by α1-GFP signal detected on native gel (Fig. 1*A*, top panel; at 30 min there was a 2 fold increase; *p* = 0.006). This is consistent with Rousseau *et. al*., although in our hands, we did not observe the strong upregulation of proteasome subunits by Western blot as they observed (38) (Fig. 1*B*). Coinciding with the increased levels of assembled proteasomes observed on native gel, there was increased hydrolysis of the suc-LLVY-AMC fluorogenic substrate after rapamycin treatment (Fig. 1*A*, lower panel; at 30 min there was a 2 fold increase; *p* = 0.03). This increase in GFP signal and activity was transient, as levels peaked at 30 to 60 min. Although cellular protein amount and composition change dramatically upon induction of general autophagy, this trend was similar irrespective of loading equal amount of proteins or equal volume of cell lysates on native gel (Fig. S2). The rapid increase in proteasome abundance was dependent on protein synthesis as the translation inhibitor cycloheximide largely prevented proteasome upregulation (Fig. S3). Although this upregulation might appear fast considering a reported maturation half-time of more than 30 min (41), other studies have shown faster CP maturation (42). The reduction in proteasome levels at later time points was accompanied by an increase in a faster-migrating GFP species on the fluorescent scan of the native gel. The accumulation of this species showed kinetics similar to the accumulation of a 25 kDa band observed on GFP immunoblots (Fig. 1*B*). This species migrated to where we anticipate free GFP would be on native gel (43). This “free GFP” is generally indicative of vacuolar targeting of GFP-tagged proteins (28, 44). We observed the same dynamics when the RP subunit Rpn1 was tagged with GFP (Fig. S4). Growth for 24 h in yeast-peptone dextrose (YPD) media, which does not induce general autophagy, did not yield such an increase in this GFP species and resulted in little to no free GFP on immunoblots compared with nitrogen starvation which induces proteasome autophagy (Fig. S5). In sum, rapamycin treatment of *Saccharomyces cerevisiae* resulted in an initial upregulation of proteasomes followed by vacuolar degradation.

To test if the vacuolar targeting upon rapamycin treatment was autophagy dependent, we introduced Rpn1-GFP or α1-GFP in the autophagy-deficient *atg7Δ* strain. No accumulation of free GFP was detected in the *atg7Δ* cells, indicating that both the proteasome RP and CP are autophagy substrates upon inhibition of TORC1 (Fig. 1*C*). Fluorescent microscopy analyses showed that proteasomes are enriched in the nucleus during logarithmic growth (45, 46), with the vacuoles devoid of fluorescence signal. This localization remained similar when cells were grown in rich media for 24 h (Fig. 1*D*). Vacuoles can often be identified with brightfield images, nevertheless, we confirmed the assignment by staining with FM4-64 (stains vacuolar membrane) or CMAC-Arg (stains vacuolar lumen) (Fig. 1*D*). Consistent with the gel analyses, 24 h post rapamycin addition, vacuolar fluorescence was observed (Fig. 1*E*). 55% of WT cells showed vacuolar localization of proteasomes after rapamycin treatment (n > 100, SEM= 1.3 from 3 independent experiments replicates), suggesting this response is less robust than nitrogen starvation, where we observed 90% of the cells with vacuolar fluorescence (28). Consistent with immunoblotting data, the lack of vacuolar fluorescence in *atg7Δ* cells indicates the translocation is autophagy dependent after rapamycin treatment. Overall, our data show that upon rapamycin treatment, proteasomes undergo a bi-phasic response where an initial upregulation in proteasome levels and activity is followed by an overall reduction via autophagy. Notably, a fraction of generally larger cells showed little to no proteaphagy with rapamycin (Fig. 1*E*, open arrowheads α1 + rapa).

### Proteaphagy is distinct from general autophagy

Nitrogen starvation and rapamycin treatment both lead to the inactivation of TORC1 with various downstream effects, such as nuclear translocation of the nitrogen responsive transcription factor Gln3 (34, 47). Thus, rapamycin and nitrogen starvation elicit, at least in part, a similar response through TORC1 inhibition. However, we observed proteasome upregulation only with rapamycin and not upon nitrogen starvation (Fig. 1) (43). This suggests that proteasomes respond differently to these two conditions that induce general autophagy. Therefore, we followed the proteasome response to other conditions and drugs that induce general autophagy.

**Figure 2:**
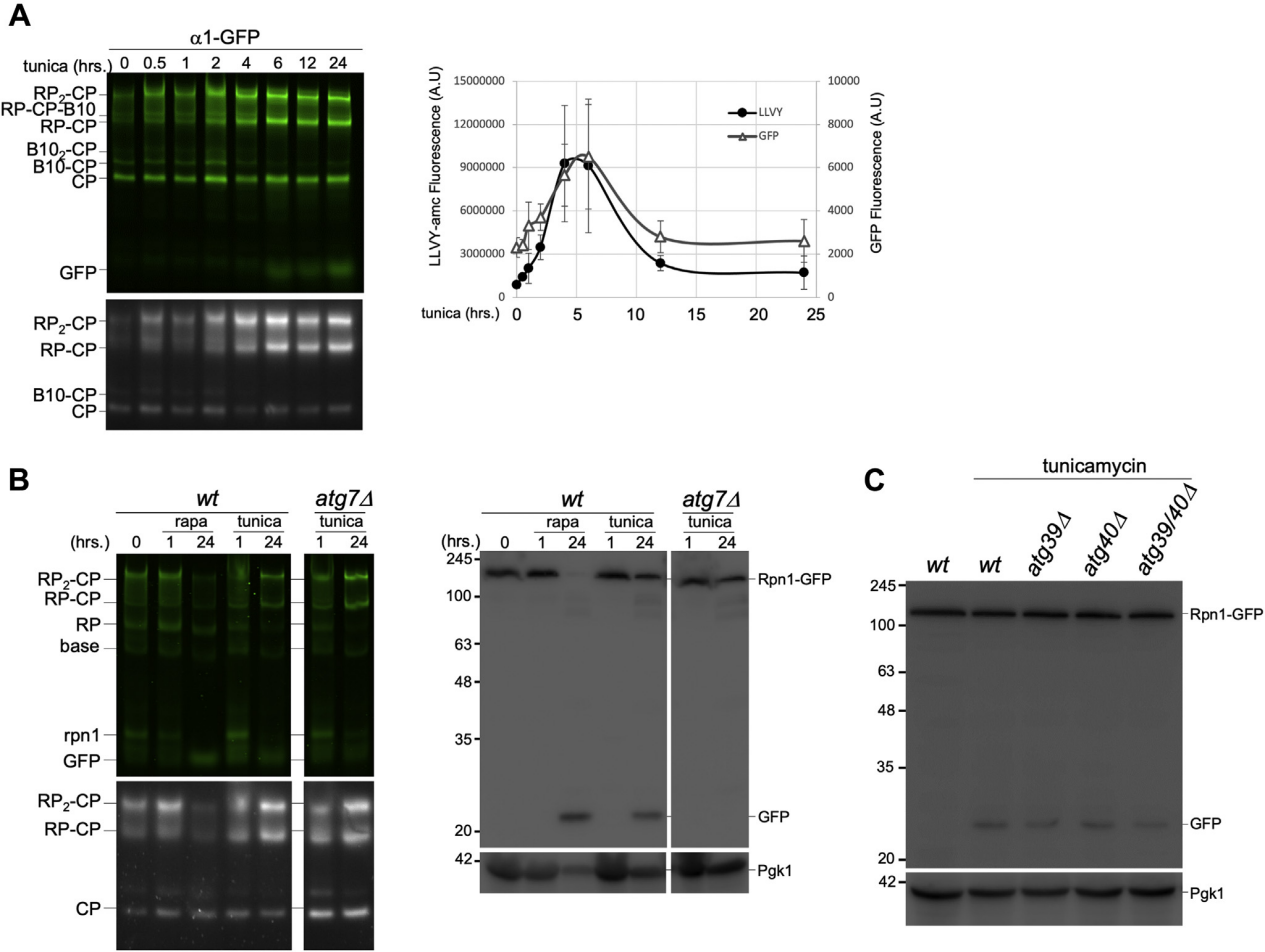
**Tunicamycin induces a bi-phasic proteasome response.***A,* yeast expressing α1-GFP were treated with tunicamycin (6 μM) for indicated time periods. The cells were collected, lysed under native conditions, and equal amounts of lysates were loaded on native gel. After separation, gels were imaged for GFP fluorescence (*top*) or suc-LLVY-AMC peptidase activity in the presence of 0.02% SDS (*bottom*). Quantifications show the amount of GFP signal and LLVY peptidase activity associated with the RP_2_-CP proteasome complexes. GFP and LLVY activity were normalized to Pgk1 intensity using SDS-PAGE immunoblots of the same samples. Three independent experiments were used and the error bars represent SEM. *B,* WT and *atg7*Δ yeast were treated with tunicamycin and the lysates analyzed as in (*A*) (*left*) and denatured for Western blotting (*right*). The data presented are representative of three independent experiments (*C*) *WT, atg39Δ, atg40Δ*, and *atg39Δ atg40Δ* yeast expressing Rpn1-GFP were treated with tunicamycin as in (*A*). Two absorbances of cells were lysed using the alkaline lysis method. The samples were separated on SDS-PAGE and immunoblotting for GFP and Pgk1 was carried out as described *above*. The blots are representative of three independent experiments. CP, core particle; RP, regulatory particle.

Tunicamycin is a drug that induces ER stress, the unfolded protein response, general autophagy, and has been shown to cause proteasome upregulation (38, 48). The cells treated with tunicamycin showed a bi-phasic response similar to rapamycin, however proteasome levels peaked at 4 to 6 h instead of 1 h post treatment (Fig. 2*A*). The increase in free GFP after tunicamycin treatment reflects the induction of proteaphagy. However, less proteaphagy occurred compared with rapamycin treatment as native gels and immunoblots showed a higher ratio of Rpn1-GFP to free GFP, and more proteasome peptidase activity was detected 24 h post tunicamycin addition (Fig. 2*B*). The accumulation of free GFP was dependent on Atg7, confirming that tunicamycin induced autophagy of proteasomes (Fig. 2*B*). Because tunicamycin induces both ER autophagy and general autophagy (49), we wondered if the autophagy of proteasomes resulted from ER-associated proteasomes that traffic to the vacuole via ER-phagy. To test this, we deleted ATG39 and ATG40, two genes required for ER/nucleophagy in yeast (23). Neither gene product was required for proteaphagy under conditions of nitrogen starvation (29, 33), and we observed no reduction in the amount of free GFP generated upon tunicamycin treatment when ATG39, ATG40, or both genes were deleted (Fig. 2*C*). Thus, proteaphagy observed upon tunicamycin treatment is distinct from ER-phagy.

We next tested another inhibitor of TORC1, caffeine (50, 51). We did not detect an upregulation of proteasome levels or activity when caffeine was added to growing cells, (Fig. S6). This difference between caffeine and rapamycin could result from differential inhibition of TORC1 downstream pathways with these drugs or TORC1-independent effects of rapamycin or caffeine. However, rapamycin is potent in yeast, and both drugs show a similar response in transcriptional profile (52, 53). Consistent with this, proteaphagy was also detected after caffeine treatment. However, free GFP accumulated slower and to a lesser extent compared with rapamycin treatment or nitrogen starvation. Caffeine is less potent as an inhibitor of TORC1 as compared with rapamycin or nitrogen starvation (50, 51), which might impact the relative magnitude and timing of TORC1 inhibition, resulting in differences in the proteasome response.

**Figure 3:**
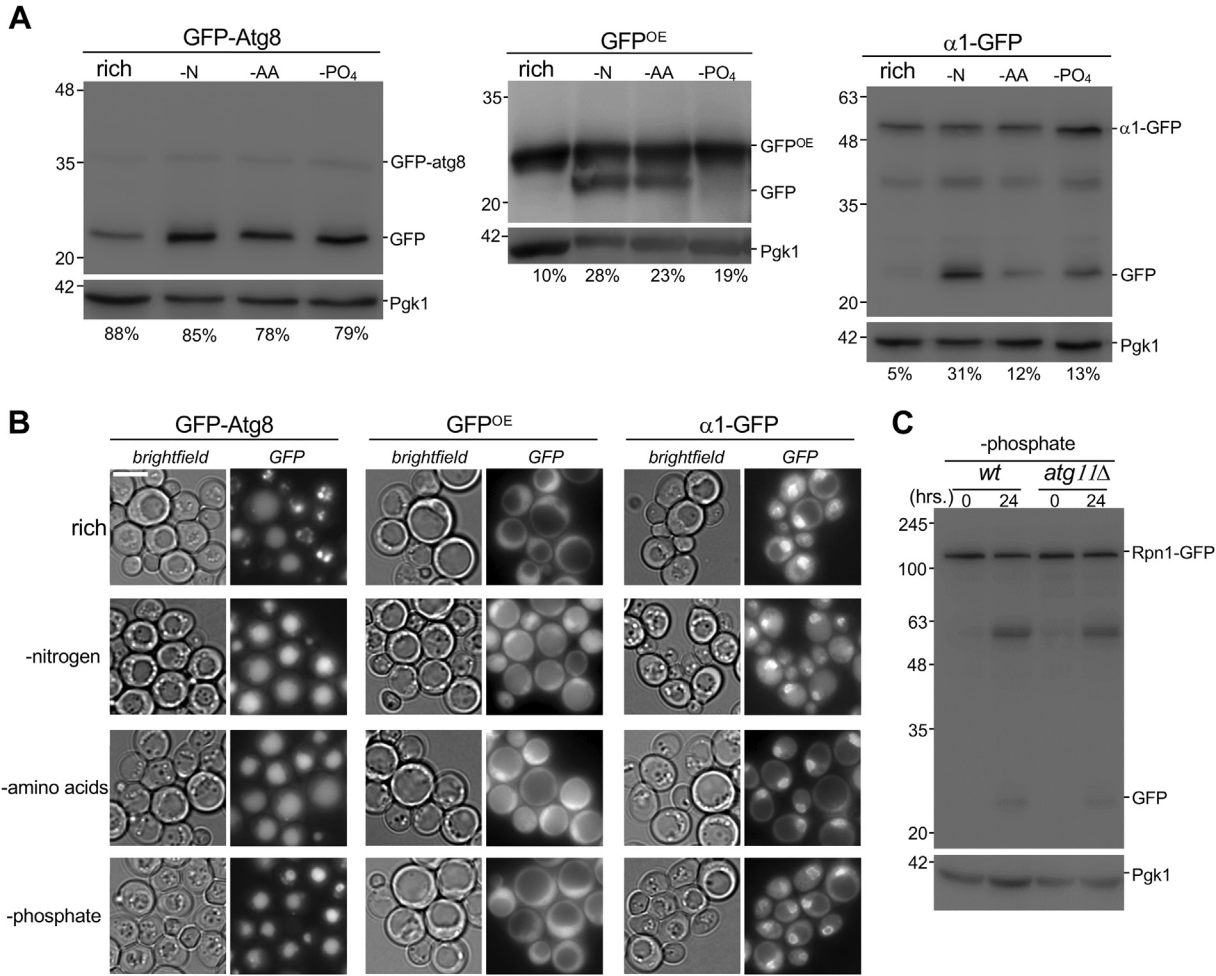
**Proteaphagy is distinct from general autophagy.***A,* yeast strains expressing GFP-atg8, GFP^OE^, or α1-GFP, were grown in YPD and subsequently starved 24 h for nitrogen (-N), amino acids (-AA), or phosphate (-PO_4_). The equivalent of 50 absorbances of cells was lysed by cryogrinding. Equal volumes of the lysate were blotted for GFP and Pgk1. The value below immunoblots indicate the free GFP signal as percentage of free + fused GFP. The blots presented are representative of three independent experiments (*B*) localization of fluorescent proteins in starved cells from (*A*) was monitored by microscopy. The scale bar represents 5 μm and data presented are representative of three independent experiments. *C, WT* and *atg11Δ* yeast were grown to log phase in rich medium and switched to phosphate-starvation medium. Two absorbances of cells were harvested at indicated timepoints and analyzed as *above*. The data presented are representative of three independent experiments. YPD, yeast-peptone dextrose.

Treating *S. cerevisiae* with the different general autophagy inducing drugs, as shown above, led to proteaphagy, although each drug elicited different responses with regard to proteasome levels and activity over time. Starving cells for different nutrients, such as nitrogen, phosphate, amino acids, or carbon, also induces general autophagy (54, 55, 56, 57). To compare general autophagy with proteaphagy under these starvation conditions, we used GFP-Atg8, which has been used to monitor bulk autophagy (58, 59). Although we detected GFP cleaved from Atg8 (indicative of vacuolar targeting) under starvation conditions, we also observed cleavage when cells were grown in rich media for the same amount of time, a condition where little to no bulk autophagy has been reported (Fig. 3*A*). Considering Atg8 is not only involved in bulk autophagy, but also a number of selective autophagy pathways such as the cytoplasm-to-vacuole targeting pathway (58) this read-out might not be ideal. Therefore, we used as a second reporter overexpressed GFP (GFP^OE^) not linked to any other protein. Being a heterologously expressed protein without specific interaction domains, GFP^OE^ should not associate with structures or complexes in the cell. Therefore, its lysosomal processing better reflects bulk autophagy. Deletion of ATG7 in the strains expressing GFP^OE^ prevented the formation of a GFP product of smaller molecular weight under starvation conditions, indicating the smaller product can be used as read-out of GFP^OE^ undergoing autophagy (Fig. S7, left panel). When grown in rich media, this reporter did not produce any significant amount of free GFP (Fig. 3*A*). As GFP^OE^ autophagy has not previously been used as an autophagy reporter, we also included cytosolic rosella as an additional control (Fig. S7, right panel) (60). When we starved cells for amino acids, we observed increased free GFP from GFP-Atg8 compared with rich medium and a similar increase as seen with nitrogen starvation (Fig. 3, *A* and *B*). This indicates comparable autophagic flux for Atg8 under these conditions, consistent with previous reports (55). Free GFP was also observed in our GFP^OE^ and cytosolic rosella strains upon amino acid or nitrogen starvation. Proteaphagy, on the other hand, was not induced to a similar extent after amino acid starvation compared with nitrogen starvation, as indicated by the reduced amount of free GFP (Fig. 3*A*).

Phosphate starvation induces general autophagy, albeit to a lesser extent than nitrogen starvation (56). Consistent with this, we found only a modest level of autophagy for GFP^OE^ and cytosolic rosella (Fig. 3*A* and Fig. S7) even though GFP-Atg8 was targeted to vacuoles robustly. This suggests phosphate starvation induces little general autophagy in our strain and the GFP-Atg8 processing we observed could be derived mainly from selective autophagy. This is supported by the reported requirement for ATG11 in phosphate starvation-induced autophagy (56), as Atg11 is a protein normally involved in selective autophagy. Proteasomes also appeared to undergo phosphate starvation induced autophagy, albeit to a lesser extent than under nitrogen starvation (Fig. 3*A*). Atg11 was not required for proteaphagy after phosphate starvation, as we did not detect a reduction in the amount of free GFP for the RP upon ATG11 deletion (Fig. 3*C*).

### Proteaphagy requires factors not involved in general autophagy

**Figure 4:**
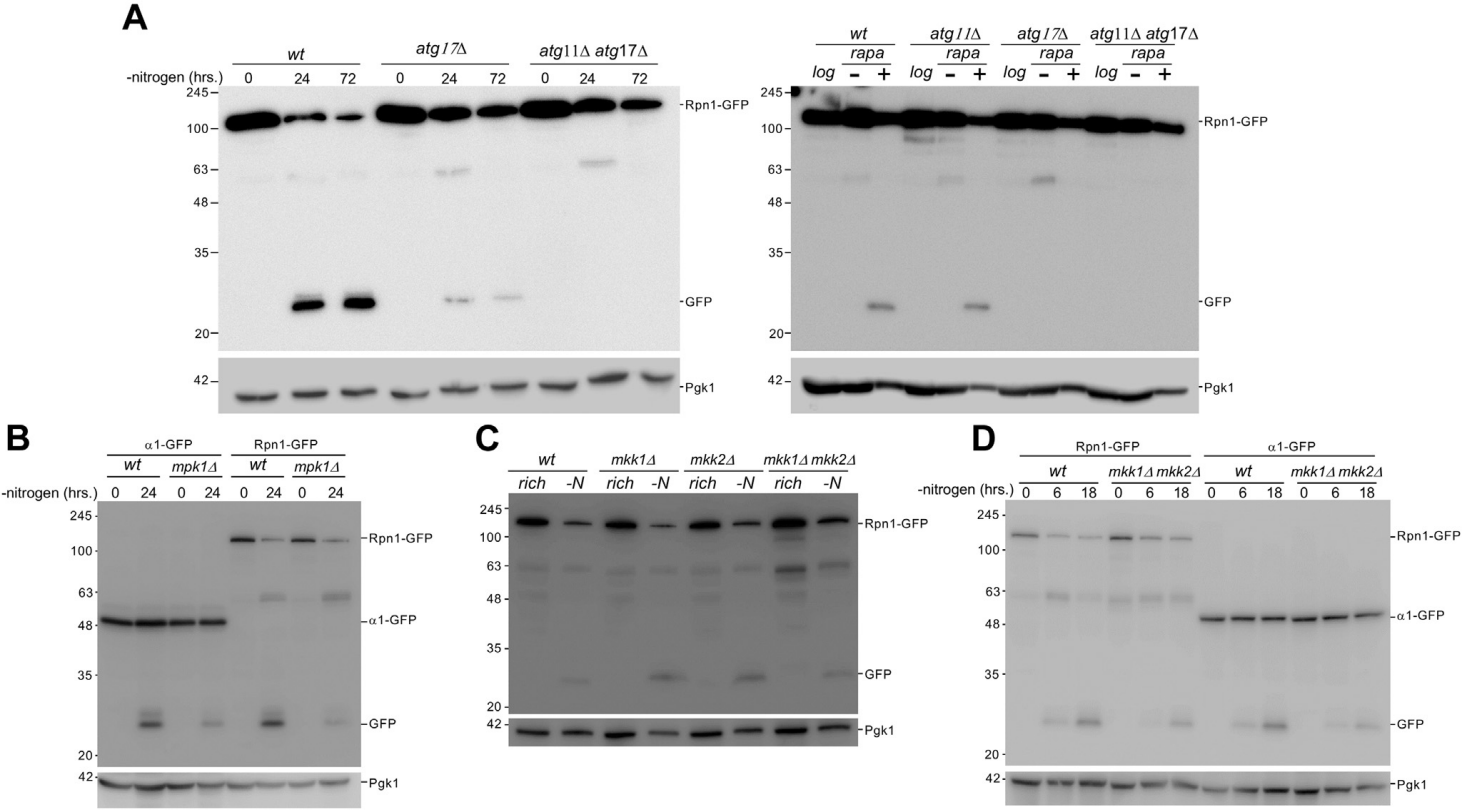
**Proteaphagy requires factors dispensable for general autophagy.***A, WT, atg11Δ, atg17Δ, atg11Δ atg17Δ* yeast expressing Rpn1-GFP were starved for nitrogen or treated with rapamycin for 24 h. The data presented are representative of three independent experiments. *B, WT* and *mpk1Δ* cells expressing Rpn1-GFP or α1-GFP were starved for nitrogen and 2 absorbances were harvested at indicated time points. Immunoblotting for GFP and Pgk1 were performed, as described *above*. We observed an ∼35% reduction in cleaved GFP from Rpn1 and ∼33% from α1 (*p* value 0.067 and 0.0005, respectively from unpaired *t* test) in the mutants compared with WT 24 h after starvation. The data presented are representative of three independent experiments. *C, WT*, *mkk1Δ, mkk2Δ,* and *mkk1Δ mkk2Δ* cells expressing Rpn1-GFP were starved for nitrogen and 2 absorbances of cells were harvested at 24 h. Immunoblotting for GFP and Pgk1 were performed, as described *above*. The data presented are representative of three independent experiments. *D, WT* and *mkk1Δ mkk2Δ* cells expressing Rpn1-GFP or α1-GFP were starved for nitrogen and 2 absorbances of cells were harvested at indicated time points. From three independent experiments, we observed an ∼39% reduction in cleaved GFP from Rpn1 and an ∼40% reduction from α1 (*p* = 0.030 and 0.119 respectively from unpaired *t* test).

The conditions used above induced general autophagy, but proteasomes were not always robustly targeted for degradation. This suggests proteasomes are not subject to general autophagy but are regulated by a selective process. On the other hand, considering the majority of proteasomes are nuclear, our results could also reflect a lag time for the targeting of nuclear proteins to general autophagy due to the need for nuclear export (Fig. 1*D*) (45, 46). A hallmark that distinguishes general autophagy from selective autophagy is that the latter pathway depends on specific receptors that recognize and sequester substrates into autophagosomes. For example, selective autophagy of peroxisomes uses the receptor Atg30, ER-phagy uses Atg39 and/or Atg40 and mitophagy Atg32 or Atg33 (10, 23). All of these factors can interact with the selective autophagy scaffolding factor Atg11 (61, 62). General autophagy, on the other hand, does not depend on Atg11 (63). We have previously reported that nitrogen starvation-induced autophagy of an Rpn1-GFP fusion strain was not entirely abolished by a deletion of *ATG17* (28). Interestingly, the residual autophagy of proteasomes in the *atg17Δ* strain was abolished after deletion of *ATG11* (Fig. 4*A*). For rapamycin-induced proteaphagy, we were unable to detect free GFP in the *atg17Δ* strain, and no difference in free GFP was detected in the *atg11Δ* strain compared with WT strain. This suggests that Atg11 is not essential for any form of proteaphagy induced by rapamycin. However, less proteaphagy is induced with this drug in general compared with nitrogen starvation, and the free GFP levels might be below our detection limit. Other factors required for proteaphagy, such as p62 in humans and Cue5 and Atg24 in yeast (30, 31, 33), further support the notion that some forms of proteaphagy proceed through a selective pathway.

To identify other factors involved in proteaphagy, we focused on a key MAP kinase signaling pathway in yeast which uses the kinase Mpk1. Mpk1 is not involved in general autophagy (64) but regulates proteasome abundance upon rapamycin addition (38). Deletion of MPK1 in our Rpn1 GFP-tagged proteasome strain resulted in ∼35% reduction in the amount of cleaved GFP (going from 62% in WT to 40% in *mpk1*Δ *p* = 0.067). When α1 was tagged with GFP, we observed a reduction of ∼33% (going from 40% in the WT to 27% in the knock out; *p* = 0.0005) (Fig. 4*B*). This indicates a role for Mpk1 in efficient proteaphagy upon nitrogen starvation. As discussed above, caffeine-induced proteasome autophagy albeit later and to a lesser extent than rapamycin or nitrogen starvation. This drug inhibits TORC1 and thereby activates the cell wall integrity (CWI) pathway (65, 66, 67). However, caffeine can also inhibit the redundant kinases Mec1 and Tel1 in the DNA damage response pathway (66). Both the CWI and DNA damage response pathways require Mpk1. To test if proteaphagy induced by caffeine required Mpk1, we exposed *mpk1*Δ cells to caffeine and monitored proteaphagy. Proteaphagy was not detectable in *mpk1*Δ cells after caffeine treatment (Fig. S8). If the DNA damage response pathway is involved, Tel1 and Mec1 would be required. As MEC1 is essential, we monitored proteaphagy induced by caffeine in WT and TEL1 deleted yeast. We did not observe a decrease in caffeine-induced proteaphagy in *tel1*Δ cells compared with WT (Fig. S9). This is consistent with the DNA damage pathway not being involved in caffeine-induced proteaphagy. However, as Mec1 and Tel1 have redundant functions, a role for the DNA damage response pathway cannot be excluded.

To determine if kinases of the CWI pathway that are not involved in DNA damage response play a role in proteaphagy, we examined the two kinases directly upstream of Mpk1, Mkk1, and Mkk2 (68). Mkk1 and Mkk2 are redundant in function and the strains deleted of either MKK1 or MKK2 displayed normal proteaphagy upon nitrogen starvation (Fig. 4*C*). However, a reduction in proteaphagy was observed in the MKK1 and MKK2 double deletion mutant (Fig. 4, *C* and *D*). For Rpn1, we observed ∼39% reduction in cleaved GFP upon deletion of MKK1 and MKK2 (going from 44% in the WT to 27% in the double knock out; *p* = 0.030). For α1 GFP-tagged strains, we observed a consistent, although not statistically significant, reduction in free GFP formed ∼40% (going from 25% in the WT to 15% in *mkk1*Δ *mkk2*Δ strain; *p* = 0.119). Together, these data indicate that Mkk1 and Mkk2 play redundant roles and, like Mpk1, are important for efficient proteaphagy. Furthermore, the involvement of these upstream kinases suggests Mpk1 involvement in proteaphagy is not regulated through the genotoxic stress response pathway. Interestingly, deletion of BCK1, which acts upstream of Mkk1 and Mkk2, did not result in a detectable decrease in proteaphagy after nitrogen starvation (Fig. S10). This might indicate the signaling does not go through the standard CWI pathway either. Indeed, Bck1 has been shown to be dispensable for Mpk1 activation in a number of conditions (68, 69, 70). Nevertheless, the identification of roles for signal transduction (MPK1 and MKK1/2) and selective autophagy (Atg11) in proteaphagy reinforces the model that proteasomes are specifically targeted for vacuolar degradation.

## Discussion

As an important regulator of many cellular processes, including the cell cycle, the UPS fulfills an essential function. Similarly, the ability to degrade proteins via autophagy is crucial during mammalian development (71). However, individual cells, like MEFs, grow and multiply without the need for autophagy (72). Similarly, yeast strains lacking the ability to perform autophagy grow at WT rates under optimal conditions. Autophagy becomes essential, however, upon exposure to certain stress conditions like nitrogen starvation (73). Although this indicates important and distinct functions for the UPS and autophagy, it has become clear in recent years that proteasomal and autophagic protein degradation can be synergistic. Here, one system can, to a certain extent, compensate for the impairment of the other (15, 18). Interestingly, in the context of this synergy, recent observations show proteasomes themselves are substrates for autophagic degradation (28, 29, 30, 31, 33). Although such degradation makes sense under conditions where proteasomes are damaged or not functional (e.g., as a result of inhibition), it is less clear why proteasomes are degraded upon nitrogen starvation. During nitrogen starvation, protein degradation through the UPS and autophagy could contribute to replenishing amino acid pools that become depleted. One possible explanation is that the translation is largely blocked during nitrogen starvation and ribosomes, which form a substantial amount of the cellular protein mass, are targeted for autophagy (24, 74). Thus, less proteasome activity would be required to degrade (e.g., misfolded) proteins. Another possibility is that the cells use highly abundant complexes, such as ribosomes and proteasomes, as a pool of resources that can be used to survive nitrogen starvation. The need for the ribophagy receptor NUFIP1 in reactivation of TORC1 signaling upon extended arginine starvation of HEK-293 cells indicates such a role for ribophagy (27).

Approximately, 70% of proteasomes in yeast are nuclear and direct autophagy of nuclear material is not responsible for vacuolar targeting of proteasomes. Instead, nuclear export is required for their efficient degradation through autophagy (28, 33). Here, we show that MAPK signaling is important for the efficient degradation of proteasomes, suggesting that specific signaling events regulate proteaphagy. A master regulator of metabolic signaling is TORC1. This kinase is inhibited under nitrogen starvation, a condition that can be mimicked with the drug rapamycin. Therefore, the observation that proteasomes are upregulated upon TORC1 inhibition was surprising (38). Our extended analyses showed that proteasomes are quickly upregulated in a translation-dependent manner (within 30 min). After upregulation, the proteasomes are degraded through autophagy. A similar bi-phasic response was also observed with tunicamycin. Because tunicamycin elicits the unfolded protein response, the upregulation of proteasomes seen with this drug seems logical as proteasomes are important for the clearance of misfolded proteins (75, 76). Consistent with this, the transcriptional regulator Rpn4, responsible for the upregulation of proteasome genes and several other genes, is required for cell survival after inducing ER stress with tunicamycin (77). Proteasome upregulation by rapamycin likely reflects a similar cellular response that benefits cell survival under specific conditions. In line with this, the lack of proteaphagy upon amino acid starvation in yeast may indicate that proteasome activity contributes to replenishment of amino acid pools. Starvation for nitrogen in yeast also leads to depleted amino acid pools (12, 78), thus the ability of proteasomal degradation to replenish amino acids might be important early in nitrogen starvation. Indeed, we observed delayed proteaphagy compared with general autophagy in yeast (starting ∼6 h versus ∼2 h) (43, 79).

Another possibility for the different dynamics of proteasomes under autophagy inducing stimuli is that early proteasome activity in autophagy induction may facilitate cellular reprogramming and stress responses. In support of this idea, TORC1 inhibition upon nutrient deprivation has recently been shown to lead to the proteasomal degradation of proteins required for DNA replication (80). In addition, degradation of translation and RNA turnover factors by the proteasome, like Pop2, a deadenylase subunit, and Dcp2, a de-capping enzyme, have been reported upon both nitrogen starvation and rapamycin treatment (74). These data support a role for proteasome activity in regulating protein degradation during autophagy. However, other factors related to ribosome function are degraded via autophagy and do not require proteasome activity (74). Thus, it appears that protein degradation after autophagy induction occurs through both the UPS and autophagy pathways, exemplifying their synergy, and facilitate the appropriate cellular rereprogramming.

The direct evidence for specificity in proteaphagy comes from the identification of specific factors that are required for proteaphagy but are dispensable for bulk autophagy. Examples are the requirement for Rpn10 in plants, Atg24 and Cue5 in yeast, and p62 in mammalian cells (29, 30, 31, 33). In the current study, we present further evidence in support of the specific proteaphagy upon nitrogen starvation. We have previously reported that a subset of the regulatory particle was still degraded in an Atg17 mutant (28). The autophagic degradation of the remaining proteasomes depended on Atg11. Atg11 is known to be required for the specific recognition of autophagic cargo in processes like mitophagy, pexophagy, and the CVT pathway (61, 62, 81, 82). Whether or not a subset of proteasomes use a selective autophagy receptor that binds Atg11 remains to be determined.

In addition to Atg11, we also observed an important role for the MAP kinase Mpk1 in proteaphagy. Importantly, Mpk1 is not involved in general autophagy (64). Currently, it is unclear if Mpk1 facilitates proteaphagy by directly phosphorylating proteasomes, regulatory factors, or is part of a longer signaling cascade that ultimately leads to proteaphagy. Intriguingly, this kinase is also required for proteasome upregulation after rapamycin addition (38). The role of Mpk1 in rapamycin-induced proteaphagy could not be determined as *mpk1*Δ cells died approximately 4 h after rapamycin addition, which is before robust proteaphagy is detectable. Mpk1 upregulates proteasomes by inducing expression of regulatory particle assembly chaperones including Adc17. Because we did not detect a role for Adc17 in proteaphagy (Fig. S11), different factors are likely involved in Mpk1's roles in the process, a subject we are currently exploring.

When mutants defective for autophagy are starved for nitrogen (28, 40) or treated with rapamycin (Fig. 1*E*), the proteasomes remain nuclear. This raises the question of how proteasome nuclear export/import is linked to autophagy flux. When autophagy is blocked by knocking out Atg7, autophagosomes fail to form (22, 83). However, cellular metabolic conditions and signals that initiate autophagy are still present in nitrogen-starved or rapamycin-treated *atg7Δ* cells. For example, in mammalian cells, the nuclear ribophagy receptor NUFIP1 still translocated to the cytosol in ATG7 deleted cells after starvation (27). Thus, proteasomes remaining nuclear suggests that the signals that govern proteasome nuclear export are not present when autophagy is blocked. The proteasome nuclear export potentially does not depend on autophagy induction but instead, autophagic flux. Consistent with this, proteaphagy is not observed until approximately 6 h post induction (Fig. 1*B*). Alternatively, it is possible that an equilibrium exists for proteasomes between the cytoplasm and the nucleus. Because only cytoplasmic proteasomes appear to be autophagy substrates, proteaphagy results in a drop in the cytosolic proteasome levels. This would induce nuclear export to maintain the nuclear-cytosolic equilibrium of proteasomes. As such, autophagy of nuclear proteasomes would be propagated passively. However, given the dependence of proteaphagy on MAP-kinase signaling, this alone does not explain why proteasomes remain nuclear since general autophagy continues. One possibility is that the nuclear-cytoplasmic equilibrium of proteasomes is maintained through cellular signaling. This suggests that the cells might regulate proteaphagy, at least for the nuclear population, by controlling nuclear export. This mechanism is different from protection against autophagy via the formation of proteasome-storage granules (84). In all, our data support a model where proteasomes are selective cargo for autophagy, regulated through MAP kinase signaling independently from general autophagy.

## Experimental procedures

### Yeast strains

All strains and oligos used in this study are reported in Tables S1 and S2 respectively. Our background strains are the W303-derived SUB61 (Matα, *lys2-801 leu2-3, and 2–112 ura3-52 his3-Δ200 trp1-1*) and SUB62 (MatA, *lys2-801 leu2-3, and 2–112 ura3-52 his3-Δ200 trp1-1*) that arose from a dissection of DF5 (85). Standard PCR-based procedures were used to delete specific genes from the genome or introduce sequences at the endogenous locus that resulted in the expression of C-terminal fusions of GFP or mCherry (86, 87, 88). GFP-atg8 expressing strains were generated by transformation with BS-Ura3-GFP-Atg8, a gift from Zhiping Xie (Addgene plasmid #69194). To create our GFP-overexpressor reporter, we generated constructs to express GFP from a GPD promotor (GFP^OE^). Plasmid pJR763 (see Table S3) was digested with Sac1 and Sal1. This produced a linear-DNA fragment for targeted integration in the *URA3-TIM9* region of the yeast genome. To identify successful integration of the expression modules in cells, the transformants were grown on plates lacking histidine. Integration was confirmed by PCR. pAS1NB c Rosella was a gift from Mark Prescott (Addgene plasmid #71245).

### Yeast growth conditions

Overnight cultures of yeast were diluted to an A_600_ of 0.5 and grown in YPD medium to an A_600_ 1.5 (approximately 4 h). The cultures were then treated with drugs for indicated time periods and harvested by centrifugation. Rapamycin (LC Laboratories) was used at 200 nM final concentration, caffeine (Sigma-Aldrich) at 10 mM, and tunicamycin (Sigma-Aldrich) at 6 μM. The translation inhibitor cycloheximide (Acros Organics) was used at a final concentration of 250 μg/ml. To induce starvation, the cultures growing logarithmically in YPD (2% dextrose) were centrifuged, washed with the respective starvation medium, reinoculated at an A_600_ of 1.5, and incubated at 30 °C with constant shaking.

### Protein lysates and electrophoresis

For native gel analyses, 50 absorbances of the cells were pelleted, washed in ddwater, and resuspended in 50 μl ddwater. This suspension was frozen dropwise in liquid nitrogen and stored at −80 °C until further processing. Protein lysates were obtained by cryogrinding cell pellets using CryoCooler and mortar and pestle from OPS Diagnostics (89). After cryogrinding, the lysates were resuspended in lysis buffer (50 mM Tris-HCl [pH 7.5], 1 mM ATP, 5 mM MgCl_2_, and 1 mM EDTA). For native gel analyses, equal volumes of lysates were loaded and separated by electrophoresis (90 V, 2.5–3 h, 4 °C). The gels were scanned on Typhoon 9410 or Trio imager (excitation at 488 nm and a 526SP emission filter). The gels were then incubated with suc-LLVY-AMC and imaged using a Syngene G-Box (UV excitation, 440 nm band-pass emission filter) to visualize peptidase activity of proteasome complexes. Samples for SDS-PAGE and western analyses were prepared by mixing equal volumes of lysates for each sample with 1/5 volume of 6× SDS sample buffer (10% SDS, 40 % glycerol, 60 mM DTT, and 345 mM Tris-HCl [pH 6.8] 0.005% bromophenol blue). For experiments not involving native analysis, 2 absorbances of cells were collected at indicated time point and stored at −80 °C. Lysis was completed using previously established alkaline lysis methods (90). After electrophoreses, the samples were transferred to PVDF membranes and immunoblotted with antibodies against indicated proteins or tags followed by the appropriate horseradish-peroxidase conjugated secondary antibodies. The antibodies used were anti-GFP (1:500; Roche, cat. nr. #11814460001) and anti-Pgk1 (1:5000; Invitrogen, cat. nr. #459250). Horseradish-peroxidase activity was visualized using the Immobilon Forte Western HRP substrate (Millipore) and the images were acquired using the G-box imaging system (Syngene) with GeneSnap software. The data shown are representative of consistently observed trends from a number of independent experiments (n indicated in figure legend).

### Fluorescence microscopy

All microscopy was done with live yeast strains where proteasome subunits were fluorescently tagged (Rpn1-GFP, α1-GFP) at their endogenous locus with expression driven by the endogenous promoter. After indicated treatments, approximately 2 absorbances of cells were pelleted, washed with PBS, then resuspended in 30 μl of PBS, and 3 μl mounted on 1% soft agar slides, as described by E. Muller (91) (https://www.youtube.com/watch?v=ZrZVbFg9NE8). All the imaging by fluorescence microscopy was carried out within 10 min after washing, to avoid the effects of prolonged incubation on slides. Routinely, the yeast vacuoles were assigned based on phase contrast images. In addition, we regularly stained vacuolar membranes with the dye FM4-64 (Invitrogen; microscope settings: excitation filter 555/28 nm and emission filter of 617/73 nm) or stained the vacuolar lumen using the dye CMAC-Arg (Invitrogen; microscope settings: excitation filter 350/50 nm and emission filter of 457/50 nm). To confirm nuclear localization, DNA was stained using Hoechst (Invitrogen; microscope settings: excitation filter 350/50 nm and emission filter of 457/50 nm). The images were acquired at room temperature using a Nikon Eclipse TE2000-S microscope at 600× magnification with a Plan Apo 60×/1.40 objective Equipped with a Retiga R3^tm^ camera (QImaging). The images were collected using Metamorph software (Molecular Devices) and analyzed using ImageJ. The data presented are representative of at least three independent experimental replicates.

## Data availability

All data are contained within the article or the Supporting Information.

## Supporting information

This article contains supporting information (60, 86, 87, 88, 92).

## Conflict of interest

The authors declare that they have no conflicts of interest with the contents of this article.
