# Supplementary material for: Proteaphagy is specifically regulated and requires factors dispensable for general autophagy
Source: J Biol Chem. 2021 Dec 14;298(1):101494. doi: 10.1016/j.jbc.2021.101494 (PMC8732087; doi:10.1016/j.jbc.2021.101494)
Supplement: Supporting information [file mmc1.pdf]

– Supplementary information –

Proteaphagy is specifically regulated and requires factors dispensable for general autophagy

Kenrick A. Waite[1], Alicia Burris[1,2,3], Gabrielle Vontz[1,4], Angelica Lang[2], and Jeroen Roelofs[1]

[1] Department of Biochemistry and Molecular Biology, University of Kansas Medical Center, Kansas City, 3901 rainbow Blvd., HLSIC 1077, KS 66160, USA

[2] Molecular, Cellular, and Developmental Biology Program, Division of Biology, Kansas State University, 338 Ackert Hall, Manhattan, KS 66506, USA

[3] Biology & Environmental Health, Missouri Southern State University, 3950 Newman Road, Joplin, MO 64801, USA

[4] Current address: Department of Genetics, Louisiana State University Health Sciences Center 1700 Tulane Avenue, New Orleans, LA 70112O, USA

Running Title: Proteasome autophagy is distinct from general autophagy

Includes:

Supplementary Figures S1 to S10

Supplementary Tables S1 to S3

Supplementary Figures

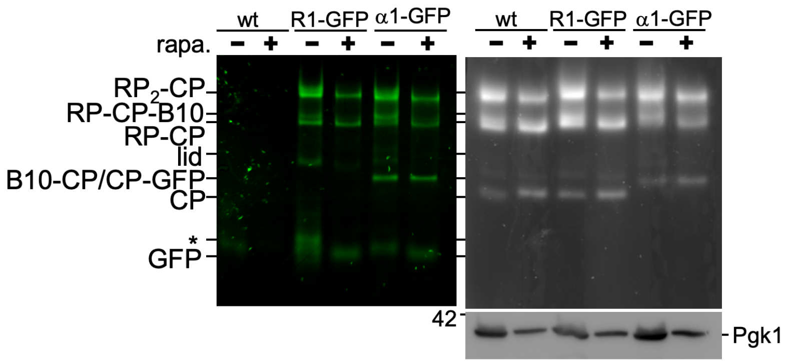

Supplementary figure 1. Wild type (wt), Rpn1-GFP, and α1-GFP yeast strains were grown in the absence or presences of rapamycin as indicated. Logarithmically grown cells were lysed by cryo-grinding and equal volumes of lysate were loaded on native gel and separated by electrophoresis. Gels were imaged for GFP fluorescence (left) and suc-LLVY-AMC peptidase activity in the presence of 0.02% SDS (right). As loading control, identical amounts of the same samples were denatured, separated by SDS_PAGE, transferred to PVDF membrane and immunoblotted for Pgk1. Data presented are representative of 2 independent experiments. (*) indicates background band as it is observed in a strain without a GFP tag.

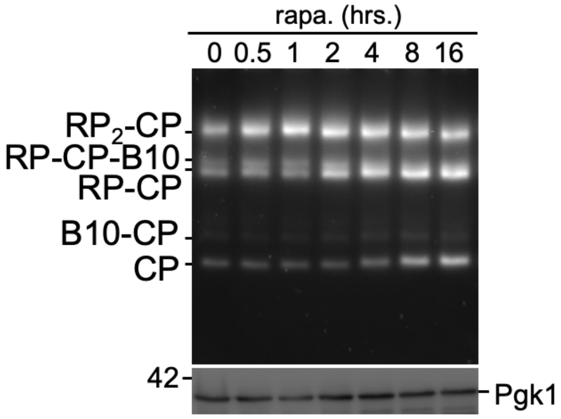

Supplementary figure 2. Wild type yeast cells were treated with rapamycin and samples were collected at indicated times. Following cryo-lysis protein concentrations were determined by nano drop and equal amount of proteins were loaded on native gel (100μg/lane). Following electrophoresis, the gel was imaged for suc-LLVY-AMC peptidase activity in the presence of 0.02% SDS. The same amount of samples were run on SDS-PAGE then transferred to PVDF membranes for immunoblotting against Pgk1 as a loading control. Data presented are representative of three independent experiments.

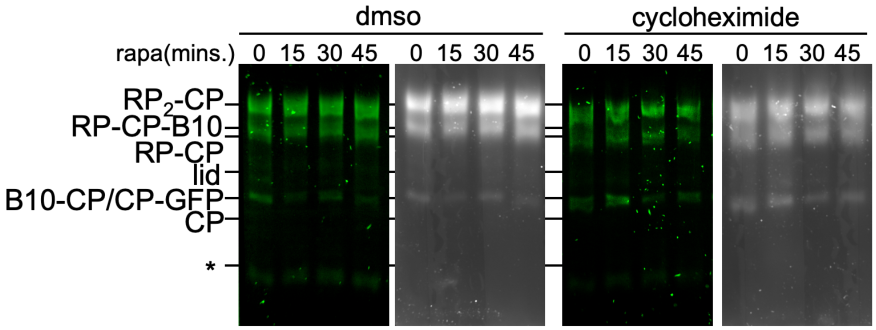

Supplementary figure 3. An α1-GFP expressing yeast strain was grown to log phase and treated with DMSO or cycloheximide for 1 hour. Next, rapamycin was added to the cells and samples collected at the indicated time points. Following native PAGE, gels were imaged for GFP fluorescence (left) and suc-LLVY-AMC peptidase activity in the presence of 0.02% SDS (right). Data presented are representative of two independent experiments. * indicates background see Supplementary fig. 1.

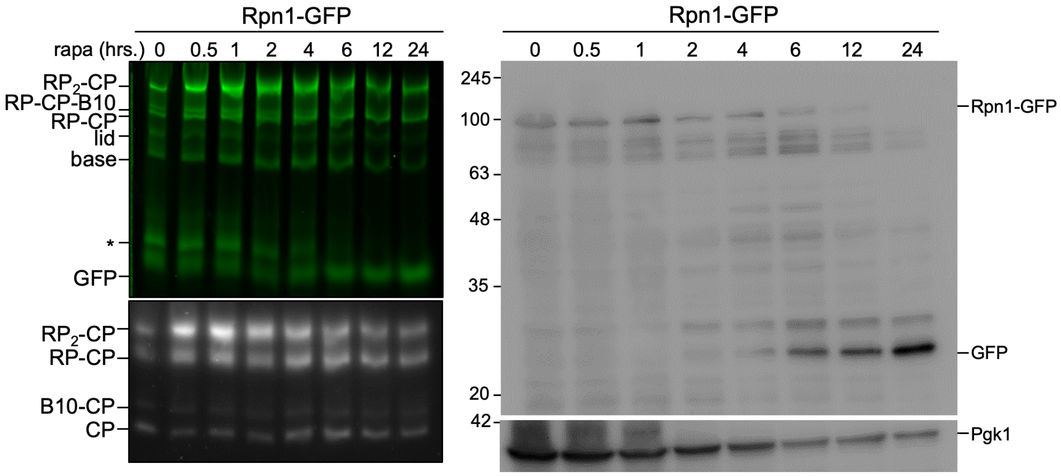

Supplementary figure 4. A strain expressing Rpn1-GFP was treated with rapamycin and samples were collected at the indicated time points. Following cryo-lysis, lysates were separated by native PAGE and imaged for GFP fluorescence and suc-LLVY-AMC peptidase activity in the presence of 0.02% SDS (left panels). * indicates background see Supplementary fig. 1. Samples were also denatured and immunoblotted for GFP and Pgk1 (right panels). Data presented are representative of three independent experiments.

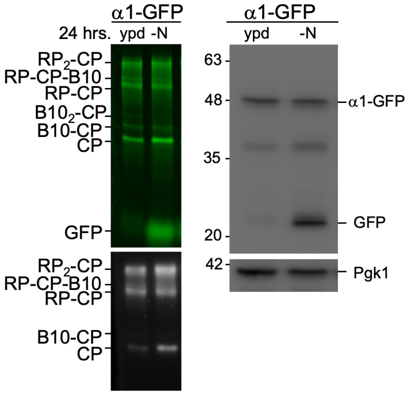

Supplementary figure 5. Yeast expressing α1-GFP were grown in YPD media or nitrogen starvation media for 24 hours. Samples were collected, lysed, and lysates were separated by native PAGE and imaged for GFP fluorescence and suc-LLVY-AMC peptidase activity in the presence of 0.02% SDS or separated by SDS-PAGE followed by immuno-blotting against GFP or Pgk1. Data presented are representative of consistently observed trends from multiple independent experiments. Please note, immunoblot complimenting the native gel analyses is part of the same blot presented in Fig. 3A, where these samples served to compare the conditions with amino acid and phosphate starvation.

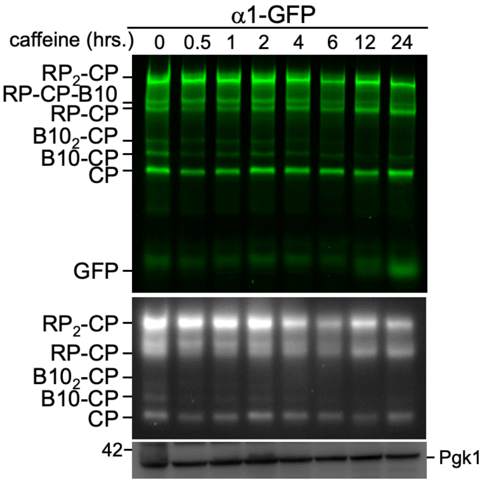

Supplementary figure 6. Yeast expressing α1-GFP were treated with caffeine, lysed, and analyzed by native gel as described above. The same amount of samples were run on SDS-PAGE then transferred to PVDF membranes for immunoblotting against Pgk1 as a loading control. Data presented are representative of three independent experiments.

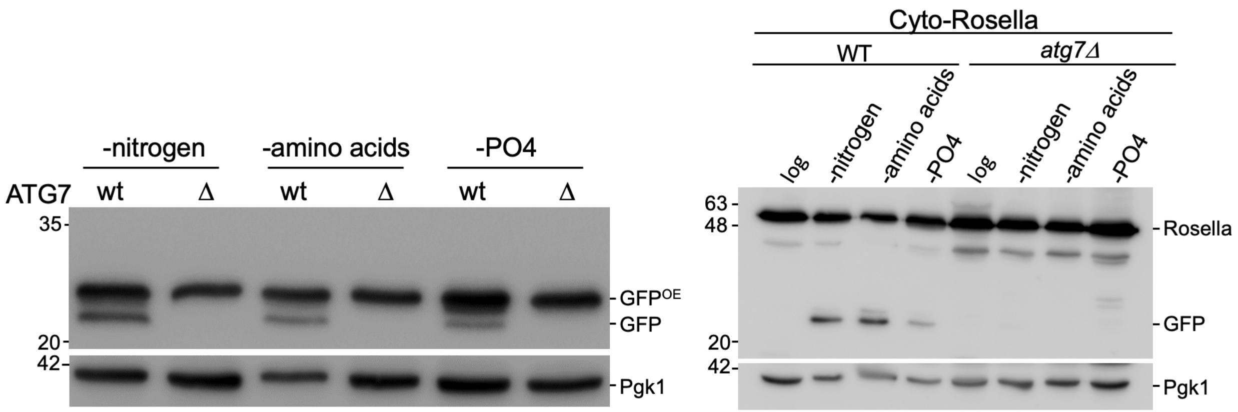

Supplementary figure 7. WT and *atg7Δ* yeast over-expressing GFP were starved for nitrogen, amino acids, or phosphate. Samples were lysed using the alkaline lysis method, separated by SDS-PAGE and immunoblotted for GFP and Pgk1 (left). WT and *atg7Δ* yeast expressing cytosolic rosella were starved for nitrogen, amino acids, or phosphate. Samples were lysed and analyzed as described for the left panel (right). Data presented are representative of three independent experiments.

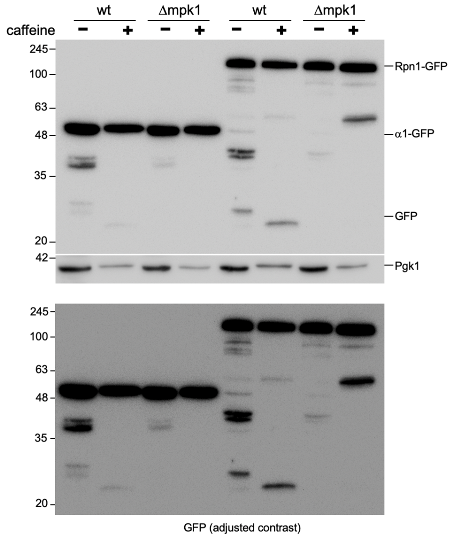

Supplementary figure 8. WT and MPK1 deleted yeast expressing Rpn1-GFP or α1-GFP were treated with vehicle (H_2O) or caffeine for 24 hours. Samples were lysed as described for supplementary figure 7 and immunoblotted for GFP and Pgk1. Data presented are representative of two independent experiments.

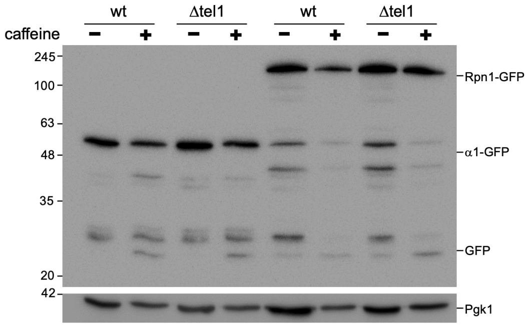

Supplementary figure 9. WT and TEL1 deleted yeast expressing Rpn1 or α1 GFP were treated with vehicle (H_2O) or caffeine for 24 hours. Samples were lysed as in supplementary figure 7 and blotted for GFP and Pgk1. Data presented are representative of two independent experiments.

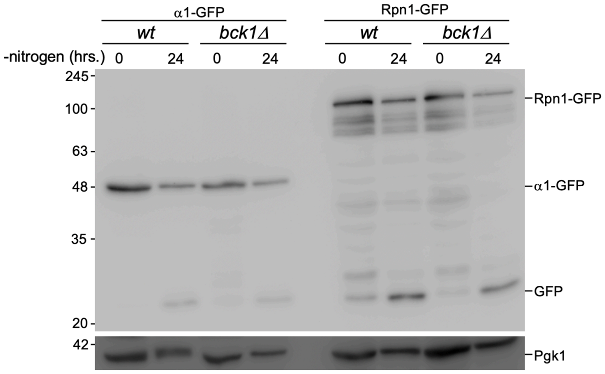

Supplementary figure 10. WT and BCK1 deleted yeast expressing α1-GFP or Rpn1-GFP were starved for nitrogen and samples were collected at 0 and 24 hours after starvation. Lysates were separated on SDS-PAGE and immunoblotted for GFP or Pgk1. Data presented are representative of three independent experiments.

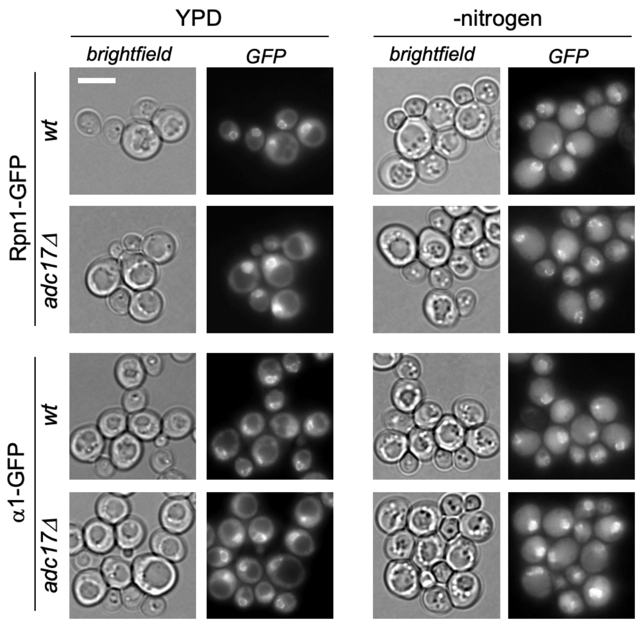

Supplementary figure 11. Wildtype and *adc17Δ* yeast expressing Rpn1-GFP or α1-GFP were grown in YPD medium or nitrogen starvation medium for 24 hours and microscopic analyses were performed. Scale bars represent 5 μm. Data presented are representative of three independent experiments.

Supplementary table S1. strains used in study.

Strain	Genotype (*lys2-801 leu2-3, 2-112 ura3-52 his3-Δ200 trp1-1*)	Figure	Source
sUB61	MAT*α*	**S2**	(1)
sJR786	MAT*α atg7::CloNat rpn1::RPN1-GFP (HIS3)*	**1c,e 2b**	(2)
sJR861	MATα *rpn1::RPN1-GFP (HIS3)*	**1c,d,e 2b,c 3c 4a,b,c,d S1,4,8,9,10,11**	(2)
sJR882	MATα *rpn1::RPN1-GFP (HIS3) atg17::HYG*	**3d 4a**	(2)
sJR900	MATα *rpn1::RPN1-GFP (HIS3) atg11::G418*	**3c 4a**	(2)
sJR923	MATα *rpn1::RPN1-GFP (HIS3) atg39::HYG*	**2c**	(4)
sJR992	MATα ura3 ^{P}Atg8-*GFP-atg8 (URA3)*	**3a,b**	(4)
sJR1035	MATα *ura3 ^{P}GDP- ubi-met-GFP (HIS5)*	**3a,b**	(4)
sJR1084	MATα *scl1::SCL1-GFP (HIS3)*	**1a,b,c,e 2a, 3a,b 4b,d S1,3,5,6,8,9,10,11**	(3)
sJR1086	MATA *atg7::CloNat scl1::SCL1-GFP (HIS3)*	**1c,e**	(3)
sJR1091	MATα *rpn1::RPN1-GFP (HIS3) atg11::G418 atg17::Ura*	**4a**	(4)
sJR1151	MATα *rpn1::RPN1-GFP (HIS3) mpk1::G418*	**4b**	(4)
sJR1216	MATα *scl1::SCL1-GFP (HIS3) mpk1::HYG*	**4b**	(4)
sJR1243	MATα *rpn1::RPN1-GFP (HIS3) atg40::G418*	**2c**	(4)
sJR1244	MATα *rpn1::RPN1-GFP (HIS3) atg39::HYG atg40::G418*	**2c**	(4)
sJR1308	MATα *rpn1::RPN1-GFP (HIS3) adc17::G418*	**S11**	(4)
sJR1309	MATα *scl1::SCL1-GFP (HIS3) adc17::G418*	**S11**	(4)
sJR1327	MATα *scl1::SCL1-GFP (HIS3) bck1::G418*	**S10**	(4)
sJR1328	MATα *rpn1::RPN1-GFP (HIS3) bck1::G418*	**S10**	(4)
sJR1387	MATα *rpn1::RPN1-GFP (HIS3) mkk2::HYG*	**4c**	(4)
sJR1390	MATα *rpn1::RPN1-GFP (HIS3) mkk1::G418*	**4c**	(4)
sJR1392	MATα *rpn1::RPN1-GFP (HIS3) mkk2::HYG mkk1::G418*	**4c,d**	(4)
sJR1393	MATα *scl1::SCL1-GFP (HIS3) mkk2::HYG mkk1::G418*	**4d**	(4)
sJR1837	MAT*α pAS1NB*	**S7**	(4)
sJR1839	MAT*α atg7::CloNat pAS1NB*	**S7**	(4)
**Strain**	**Genotype (*his3Δ1 leu2Δ0 lys2Δ0 ura3Δ0*)**	**Figure**	**Source**
sJR1865	BY4742 *scl1:: SCL1-GFP (HIS3)*	**S9**	(4)
sJR1866	BY4742 *rpn1::Rpn1-GFP (HIS3)*	**S9**	(4)
sJR1867	BY4742 *tel1::G418 scl1:: SCL1-GFP (HIS3)*	**S9**	(4)
sJR1868	BY4742 *tel1::G418 rpn1::Rpn1-GFP (HIS3)*	**S9**	(4)

1. Finley, D., Ozkaynak, E., and Varshavsky, A. (1987) Cell 48, 1035-104

2. Waite, K.A., De La Mota-Peynado, A., Vontz, G., and Roelofs, J. (2015) JBC M115.699124

3. Waite, K. A., Burris, A., and Roelofs, J. (2020).. *Sci. Rep.* 10.1038/s41598-020-75126-1

4. This study

Supplementary table S2. Primers used in this study.

Primer	Genotype	Template	Sequence (5' to 3')
pRL236	*atg7::CloNAT*	pAG25[1]	TTCATTATATTTCAACAAATATAAGATAATCAAGAATAAACGTAC GCTGCAGGTCGACG
pRL237	*atg7::CloNAT*	pAG25[1]	CGGAAAGTGGCACCACAATATGTACCAATGCTATTATATGCAATC GATGAATTCGAGCTCG
pRL293	*atg17::HYG*	pFA6a-hphNT1[2]	ATTCGATACTGCGAGGATATTATCAACGTATTTAACACCTCGTAC GCTGCAGGTCGAC
pRL294	*atg17::HYG*	pFA6a-hphNT1[2]	GATACAATTATTGAATCTTTGTACCGTATCCTTTTTTTCCTATCGA TGAATTCGAGCTCG
pRL340	*atg11::G418*	pFA6a-hphNT1[2]	GTTGTTCGGAAAGTACTTCTTTTATTTTCTTTTATACATCCGTACG CTGCAGGTCGAC
pRL341	*atg11:: G418*	pFA6a-kanMX6[2]	ACATAATTAAAATCTTGTCATTTGTGACAAACGTTTAGCACATCG ATGAATTCGAGCTCG
pRL371	*atg39::HYG*	pFA6a-kanMX6[2]	TAATAGAGACTAGTAAAACAGTCGAGTTGTCGGACCTAAACGTA CGCTGCAGGTCGAC
pRL372	*atg39::HYG*	pFA6a-hphNT1[2]	CTTTTGTTAATTTCATTCTTCATGCTGGGTTTTGGATGATATCGAT GAATTCGAGCTCG
pRL451	*mpk1::G418*	pFA6a-kanMX6	GTAGAAATAATTGAAGGGCGTGTATAACAATTCTGGGAGCGTAC GCTGCAGGTCGAC
pRL452	*mpk1::G418*	pFA6a-kanMX6	GCTTACATCTATGGTGATTCTATACTTCCCCGGTTACTTATAGATC GATGAATTCGAGCTCG
pRL690	*atg40::G418*	pFA6a-kanMX6	ACGTTCTTTCTGCTGTGCTTCACTCCACCATAGAAAACTACGTAC GCTGCAGGTCGACG
pRL691	*atg40::G418*	pFA6a-kanMX6	CTTCATAGACTACCATTATGGTAAAATGGAAAAACTATTCATCGA TGAATTCGAGCTCG
pRL694	*bck1::G418*	pFA6a-kanMX6	CACTAAATATAGTATTAAAATAGTTCAACTCCACCTCCAACGTAC GCTGCAGGTCGACG
pRL695	*bck1::G418*	pFA6a-kanMX6	CGTATGCATAAATATCTTAAGTATAGATCGATCCTAATAGATCGA TGAATTCGAGCTCG
pRL718	*adc17::G418*	pFA6a-kanMX6	TAAAGCAAATCAAAACATAATAACTACTACAAGTAACATACGTA CGCTGCAGGTCGACG
pRL719	*adc17::G418*	pFA6a-kanMX6	TGACGTGAAAATGATGCGCAGTAAAACTAAATCCCGTCTCATCG ATGAATTCGAGCTCG
pRL792	*mkk2::HYG*	pFA6a-hphNT1[2]	GTTATCATATCTACAAAATACCAATTATATACACAGGATACGTAC GCTGCAGGTCGACG
pRL793	*mkk2::HYG*	pFA6a-hphNT1[2]	AAAAAGTCAGTTCTGGTTACGAGAGGAAAATGTTGGAAGTATCG ATGAATTCGAGCTCG
pRL796	*mkk1::G418*	pFA6a-kanMX6	CCAAATTAACTCTATCCTTCCATTGCACAATTTGCCCAGTCGTAC GCTGCAGGTCGACG
pRL797	*mkk1::G418*	pFA6a-kanMX6	AAAATAAACTTAATCATGTTCGCAAAAAATTGCTTATTGAATCGA TGAATTCGAGCTCG

1. Goldstein, A. L. & McCusker, J. H. Three new dominant drug resistance cassettes for gene disruption in Saccharomyces cerevisiae. Yeast 15, 1541–53 (1999).
2. Janke, C. et al. A versatile toolbox for PCR-based tagging of yeast genes: new fluorescent proteins, more markers and promoter substitution cassettes. Yeast 21, 947–62 (2004).
3. Hailey DW, Davis TN, Muller EG. Fluorescence resonance energy transfer using color variants of green fluorescent protein. Methods Enzymol. 351:34-49 (2002.

Supplementary table S3. Plasmids used in this study

Plasmid	Parent	Expression	Ref
pNC1125			(1)
pJR763	pNC1125	Ura3-GDP-UBIMdkGFP-SpHis5-TIM9	(3)
pAS1NB		Cytosolic rosella	(2)

1) Houser, John R. et al. 2012. “An Improved Short-Lived Fluorescent Protein Transcriptional Reporter for Saccharomyces Cerevisiae.” Yeast 29(12): 519–30.
2) Rosado, Carlos J. et al. 2008. “Rosella: A Fluorescent PH-Biosensor for Reporting Vacuolar Turnover of Cytosol and Organelles in Yeast.” Autophagy 4(2).
3) This study
